# Obstetrics, Gynecology and Pelvic Surgery

**Published:** 1897-05

**Authors:** William Warren Potter

**Affiliations:** Buffalo, N. Y.; Examiner in obstetrics. New York State Medical Examining and Licensing Board


### OBSTETRICS, GYNECOLOGY AND PELVIC SURGERY.

CONDUCTED BY WILLIAM WARREN POTTER, M. D., Buffalo, N. Y.
Examiner in obstetrics, New York State Medical Examining and Licensing Board.

### SHALL HYSTERECTOMY BE PERFORMED IN INFLAMMATORY DISEASES OF THE PELVIC ORGANS?

DUNNING (*Am. Jour. Obstet.*, October, 1896,) reviews this subject with the following conclusions:

1. We recognise the utility of hysterectomy in a small percentage of cases of bilateral suppuration of the tubes and ovaries in which the uterus is distinctly septic, and in cases of septic uteri which cannot be cured by other means after bilateral salpingo-oöphorectomy.

2. We oppose hysterectomy, as a rule, in inflammatory diseases of the pelvic tissues, upon the following grounds, viz.:

(*a*) The uterus is the central organ of the reproductive system, and should not, except for palpable and urgent cause, be extirpated.

(*b*) It is only in rare cases that the uterus is so far diseased as to resist the curative effect of appropriate treatment.

(*c*) The removal of the uterus profoundly affects the nervous system and emotional nature of young women deprived of this organ.

(*d*) We oppose the removal of the uterus from anatomical reasons—to-wit: As a result the vagina is shortened; the anatomical relations of the bladder, sigmoid and rectum are changed; the elasticity of the pelvic diaphragm is greatly diminished or entirely removed, the elastic tissue being largely replaced by sensitive scar tissue.

(*e*) In married women it often disturbs the sexual relations of husband and wife, and is apt to induce mental depression.

(*f*) Vaginal hysterectomy compels the use of drainage, because of the necrosis of tissue and suppuration induced.—*Memphis Med. Monthly*.

---

### HYDRAMNIOS AND ITS COMPLICATIONS.

DR. A. P. STONER (*Med. Standard*) contributes a study of this subject to the Medical Record, in which he makes the following suggestions: (1) That cases of hydramnios are frequently complicated with cardiac or renal disease, in which case the life of the fetus must take secondary consideration. (2) That faulty presentations frequently accompany this anomaly and should be sought out early. (3) That malformations of the fetus may exist and obstruct the progress to delivery. (4) That the danger of post partum hemorrhage is great and should be carefully guarded against. (5) That an antiseptic management is a prerequisite to the successful treatment of these cases.

---

Dr. Goldspohn, of Chicago, (*Dunglison's Coll. and Clin. Record,*) thinks that a certain amount of laceration of the cervix is attendant upon natural labor. If the tear does not extend more than midway between the external and internal os, it is not pathological and should be left alone; if it extends higher up, it leads to ectropion and often retroversion and demands operative measures.

---

### FERRIPYRINE.

Dr. O. Schaeffer, (*Münchener Med. Wochenschrift,* 1895, No. 53,) of Heidelberg, proposes this new hemostatic for gynecologists and obstetricians. He reports a clinical case and defines the limits of its employment as compared with ferric chloride as follows:

1. As simple hemostatic in all genital hemorrhages, in which a corrosive action is not required, as in the above case. In the parenchymatous bleeding accompanying operations the use of such a styptic in sterilised form is advantageous.

A corrosive action is only desired in the treatment of endometritis in order to destroy the remains of the infected mucous membrane after curettage, and for this purpose ferric chloride is convenient.

The styptic action is usually developed by the use of the 16 per cent. aqueous solution of ferripyrine (a beautiful deep-red liquid) with a padded sound.

2. As hemostatic and astringent, for intrauterine and vaginal injections and douches in endometritis (not only the hemorrhagic form) to give a new tone to the mucous membrane; also in metritis to excite increased secretion.

The vaginal irrigations not only influence any colpitis present, even in gonorrhea, by their astringent action, but also the exposed and hyperemic parts of the mucous membrane of the cervix, which are depleted of blood by the action of the ferripyrine, whilst the ejection of the uterine secretions is favored by the promotion of contractions.

A further desirable property, especially in vaginitis, ectropion and endometritis is the analgesic action of ferripyrine.

The employment of 1 to 1½ per cent. solutions by means of enemas or tampons is usually sufficient for these purposes or in florid, extremely painful conditions of vaginitis gonorrhoica, by insufflation of the powder.

3. The applicability of ferripyrine in powder form is a great advantage over ferric chloride. Apart from the above affections, it is consequently available for application to bleeding and extremely painful carcinoma past operation and with copious secretion. Especially is this the case when palliative surgical measures are no longer possible because the destructive ulceration is too far advanced.

The contemporaneous analgesic action is due to the relation of ferripyrine to antipyrine. Up to the present there was no powder which simultaneously stayed both blood and pain (through anesthesia), reduced the secretions and possessed no corrosive action.

The application is made by simply dusting or by mixing with fine charcoal in small gauze bags.

4. The remedy can be injected into the bladder in cases of hematuria with as little danger as into the uterus, especially as tenesmus is lessened.

For this purpose, also, 1 to 16 per cent. solutions are available, whilst liq. ferri perchlor. can only be used 1 to 800.

5. Finally, ferripyrine can be administered in much larger doses per os than liq. ferri perchlor., and especially is indicated in melena neonatorum, apart from other hemorrhages of the stomach and intestines.

The exhibition should be made in three to five grain doses for children (eight grains is the average dose for adults), prescribed with sugar and menthol, fifteen parts sugar to one part menthol.

I intend to continue the use of ferripyrine and by these lines would draw the attention of colleagues to the specific action and advantages of the drug.

---

### SEXUAL RELATION OF THE THYROID GLAND.

Fischer, in a recent paper (*Lancet*), comments upon the relations between the thyroid gland and the female sexual organs, which have often been discussed since attention was directed to the fact that goitre predominates in women—that from 80 to 90 per cent. of the cases occur in females. Myxedema, moreover, is met with in women in 86 per cent. of the cases. The same is true of Graves's disease, and in respect of sclerodermatic processes the proportion as between women and men is three to one. Swelling of the thyroid gland has been observed in puberty and at the commencement of the menstrual period. Freund found an increase of this gland during pregnancy in forty-five out of fifty cases, and Wolfler has described a case in which during pregnancy the trachea became soft and the goitre increased. Goitre increases during delivery, and decreases again some days afterward. Graves's disease also, if it develop during pregnancy, improves after delivery. It is doubtful whether the thyroid gland becomes larger during lactation, but in the climacteric period it undergoes atrophy. Goitre and phthisis seem to be incompatible, and phthisis is but rarely observed in the subjects of hypertrophied thyroid gland. Fischer next refers to the numerous theories as to the etiology of goitre: Morgagni supposed there was a communication between the thyroid gland and the larynx, and believed the trachea was moistened by this gland and phonation thereby facilitated. Some authors have suggested that this gland was a regulator of the brain, but Freund considers that it is in connection with the genitals through the sanguineo-vascular system. In the lower animals extirpation of the thyroid gland causes atrophy of the sexual organs, which is due, according to Hofmeister, to shriveling of the ovarian follicles. Hens with hypertrophied thyroid glands also lay larger eggs than those which have atrophied thyroids. Post mortem examination of a girl, aged eight years, who was affected with myxedema, showed that both ovaries were of the size usual at two years of age, and that they contained only a few follicles. The symptoms of Graves's disease depend in some degree on the condition of the patient's sexual development.—*Kansas Med. Jour.*

---

### AN OPERATION FOR THE CURE OF INCONTINENCE OF URINE IN THE FEMALE.

Gilliam (*American Journal of Obstetrics*) reports four cures of aggravated cases of the above distressing complaint by a new operation: three occurring in his own practice, one in that of his colleague, Professor Thomas C. Hoover. The operation was suggested to Gilliam by an examination of a girl of eighteen years who had suffered from incontinence from infancy, and in whom he found an anomalous band attached to the urethra near the meatus, and spreading itself over the muscles of the anterior aspect of the vulvo-vaginal junction. This was clipped with the scissors, and the incontinence immediately disappeared. In other cases he failed to find the band, and not having fully appreciated what he now considers the etiological factor of the condition, failed to cure the cases ; but, finally, after examining another patient, a girl of twenty-one, who had been through an infinity of treatment for incontinence, and failing to find any local cause, it occurred to him to free the urethra margins from the surrounding tissues. On placing her under an anesthetic, he found a membrane which occupied the position of the anterior segment of the hymen and was attached to the sides and under surface of the urethra. When put upon the stretch, it presented the appearance of wings. This membrane was excised, the excision was carried up along the urethra on either side for a distance of a third of an inch, and the raw surfaces closed by suture. The result was an immediate and complete cure. In Professor Hoover's case, there was a growth over the urethra, which was dissected out and resulted in a cure. Gilliam quotes another who has cured cases by making an incision on either side of meatus and introducing sutures parallel to the line of incision in such a way as to bring the line of union at right angles to that of the incision. The intention of this operation was to form a buttress of tissue on either side of the meatus so as to close it by mechanical pressure, but Gilliam believes that this operation, which was essentially that which he had performed, was effective as removing a source of reflex irritation, and not by a mechanical process. He quotes another case of extremely bad nocturnal enuresis in an adult in which his operation was equally successful.—*Med. Age and Kan. Med. Jour.*

---

### SOCIAL PURITY AND MARRIAGE.

E. S. Bullock, New York, says (*N. Y. Polyclinic*) that new ideas and principles increase in number in a direct ratio to the lengthening age of the world. One which can be referred to the rapid spread of scientific knowledge among the people in general, and especially to the higher education of women, is the growing conviction on the part of the gentler sex that they should not receive into their arms the men aspiring to conjugal felicity who cannot bring to them a guarantee of freedom from diseases which, when existent, may menace the health of the wife and her children and destroy the happiness which marriage should bring to all who participate in it. That great educator, the modern novel, its co-worker the problem play, and, last but not least, the higher education of women, have all been potent influences tending to take sexual relations from its place among the mysteries of life, from the darkness with which it has always been environed, and allow the clear sunlight of scientific knowledge to shine upon it. Such influences as these are to many maudlin occupants of the pulpit proofs of degeneracy; or what they are pleased to term the impure spirit of the age. To the calm and careful thinking sociologist they are evidences of a wish to see the question settled. To end controversies is preëminently the spirit of the century in which we live, and what our sexual relations should be is but one of the problems of which we seek the solution. Knowledge is not impurity; innocence which knows not its place in the world is degrading. Normal, healthful sexual intercourse is no more wrong than is the performance of any other purely physical act, and it only becomes wrong when performed in the face of social law. Our social system puts clearly defined limits upon sexual indulgence and proscribes license. Our intelligent women are waking to the fact that the social law was not made for women alone, but applies in all equity to the opposite sex as well.

A moral nature so finely developed that it will not permit its owner to enter into the marriage state, bearing the seeds of possible direful results to wife and family, is not common among men. Sometimes mistaken and pitiable marriages are the result of ignorance rather than of moral deficiency, but the family physician and the consultant gynecologist are no longer the only ones who know why such and such a wife is an invalid, or why such another one is sterile. Keen-sighted women are coming to understand these things. Victims and sufferers perhaps themselves, they are rising in defense of the daughter's health and happiness from the results of a life of lasciviousness antedating marriage on the part of the man who desires to share the conjugal bed.

From Paris, that star in the firmament of cities, as well as the cloaca of civilisation, comes the latest instance of this awakening on the part of thinking women. At a Woman's Congress recently held, resolutions were adopted to the effect that "all families must secure certificates of health from intended sons-in-law, in order to guard the daughters of the Republic from the risk of contagious or hereditary maladies in the aspiring fathers of a latter generation."

Medical men have accomplished much in promulgating the doctrines founded upon their work and study, and the world is bound to awaken to the importance of the subject when it understands that 15 per cent. of all diseases of women, excluding prostitutes, are caused by gonorrhea and its sequelæ. Still, and in spite of this, only a small proportion of the credit of educating upon these lines belongs to the medical profession. Medical literature is peculiarly barren of knowledge relating to sexual subjects. Medicine, like parents, leaves this as the one subject to be avoided.

Let us say all we can in encouragement of the women of France in their good work, and extend a welcome to the fast approaching time when the young couple starting out on the way of married life, come to each other with a clean bill of sexual health. It will be one great step toward the future marriage, which, like good life insurance, will be impossible without a certificate of freedom from hereditary or acquired disease.

---

### REPORT OF A CASE OF CESAREAN SECTION.

GEO. HAVEN (*Boston Medical and Surgical Journal*, April 30, 1896, p. 436). The case reported was that of a patient twenty-two years of age, with a justo-minor pelvis of the following measurements : anterior superior spines eight inches, crests nine inches, external conjugate six and three-quarter inches, internal conjugate four and a quarter inches, true conjugate about three and a half inches. The patient was in labor from 9 o'clock on Tuesday morning until 7.45 the following morning ; axis traction forceps were applied several times, but with no success, so, finally, the Cesarean operation, after the method of Sänger, was performed. An uninterrupted convalescence followed.

---

### DISEASED BREAST REPLACED BY LIPOMA.

THE *New York Medical Journal*, of January 30, 1897 (*Am. Jour. Surg. and Gynec.*), contains an account, taken from the *Archiv für Klinische Chirurgie*, of the removal of a diseased breast and the taking of a fatty tumor from the patient's back and grafting it into the cavity made by the excision of the mammary gland. A year later the lipoma still retained its original size, and had absolutely the fullness and appearance of the breast of the opposite side. The operation was done by Czerny, of Heidelberg.

